# Cell-Free Osteochondral Scaffold for the Treatment of Focal Articular Cartilage Defects in Early Knee OA: 5 Years’ Follow-Up Results

**DOI:** 10.3390/jcm8111978

**Published:** 2019-11-14

**Authors:** Andrea Sessa, Luca Andriolo, Alessandro Di Martino, Iacopo Romandini, Roberto De Filippis, Stefano Zaffagnini, Giuseppe Filardo

**Affiliations:** 1 Ortopedia Bentivoglio, IRCCS Istituto Ortopedico Rizzoli, 40136 Bologna, Italy; a.sessa86@gmail.com; 2 Clinica Ortopedica e Traumatologica II, Applied Translational Research (ATR) Center, IRCCS Istituto Ortopedico Rizzoli, 40136 Bologna, Italy; lucas.andriolo@gmail.com; 3 Clinica Ortopedica e Traumatologica II, IRCCS Istituto Ortopedico Rizzoli, 40136 Bologna, Italy; iacoporoma@gmail.com (I.R.); robertodefilippis88@gmail.com (R.D.F.); s.zaffagnini@biomec.ior.it (S.Z.); 4 Applied Translational Research (ATR) Center, IRCCS Istituto Ortopedico Rizzoli, 40136 Bologna, Italy; g.filardo@biomec.ior.it

**Keywords:** early osteoarthritis, surgical treatment, knee, scaffold, osteochondral, cartilage

## Abstract

The purpose of this study was to investigate the clinical results at five years’ follow-up of a tri-layered nanostructured biomimetic osteochondral scaffold used for focal articular cartilage defects in patients meeting the criteria of early osteoarthritis (EOA). The study population comprised 22 patients (mean age: 39 years), prospectively assessed before surgery, at 24 and 60 months’ follow-up. Inclusion criteria were: at least two episodes of knee pain for more than 10 days in the last year, Kellgren-Lawrence OA grade 0, I or II and arthroscopic or MRI findings according to the European Society of Sports Traumatology, Knee Surgery & Arthroscopy (ESSKA) criteria. Clinical results demonstrated significant improvement in International Knee Documentation Committee (IKDC) subjective and objective scores and in Tegner score, although activity level never reached the pre-injury level. The complication rate of this study was 8.3%. Two patients underwent re-operation (8.3%), while a comprehensive definition of failure (including both surgical and clinical criteria) identified four failed patients (16.6%) at this mid-term follow-up evaluation. The use of a free-cell osteochondral scaffold represented a safe and valid alternative for the treatment of focal articular cartilage defects in the setting of an EOA, and was able to permit a significant clinical improvement and stable outcome with low complication and failure rates.

## 1. Introduction

Osteoarthritis (OA) is a widespread orthopaedic disease [1] that is typically triggered by aging, and it has a chronic development that eventually leads to pain and functional limitation of the affected joint. However, it is not rare to encounter early OA in young patients. “Early OA” was first described by Luyten et al. [2] as “at least two episodes of joint pain for more than 10 days in the last year, radiographic Kellgren–Lawrence classification up to grade 2, and arthroscopic findings of International Cartilage Regeneration Society (ICRS) cartilage defects grades III or IV with softening and swelling of the surrounding cartilage”. Patients with early OA, often younger than those with advanced OA but still presenting a degenerated knee, represent a challenge for orthopedic surgeons since young age—with its related higher functional requests and long life expectancy—is a major issue for knee replacement. In this light, an increasing interest has recently been given to the distinction of OA in its early phases. Indeed, young patients could benefit from new biological or regenerative treatments in order to restore the damaged joint surface, thus delaying the need for more invasive conventional procedures such as arthroplasty.

The first challenge for this surgical strategy, aimed at delaying metal resurfacing, consists of a combined biomechanical and biological approach. The surgeon should identify and correct any comorbidity, which lead to the progress of degenerative changes, in order to improve the chances of success of joint surface restoration. Moreover, the OA environment is challenging per se, and it has been shown that conventional chondral and regenerative treatments offer inferior results in this setting [3,4]. A possible explanation for these somehow disappointing outcomes may be found in the intra-articular modifications taking place in OA processes, such as synovitis, matrix deterioration and subchondral bone alterations, which negatively influence tissue regeneration. Since OA degeneration involves both chondral surface and underlying subchondral bone, it has been hypothesized that an approach designed for the entire osteochondral unit could be more effective. An osteochondral biphasic scaffold, developed to induce in situ regeneration of the entire osteochondral unit, showed good preclinical and clinical results without the need for cell augmentation [5,6]. This device was successfully tested in several clinical studies [7,8,9,10] with promising results, and was proven to be stable up to mid-term follow-up. Satisfactory outcome was reported for treating complex knee articular defects and sequelae of fractures of the tibial plateau [11], as well as in cases of osteonecrosis of the distal femur [12], and promising short-term results were even highlighted in early OA joints [13]. However, only preliminary findings with short-term follow-up are available to date, and the stability of the initial improvement remains to be proven.

Thus, the purpose of this study was to determine the clinical and functional outcome of a cell-free osteochondral scaffold in terms of outcome and stability of the development up to a mid-term follow-up in a group of patients affected by focal articular cartilage defects in early OA knees.

## 2. Materials and Methods

This study was authorized by the Hospital Ethics Committee and Internal Review Board, and informed consent was acquired from each patient.

The inclusion criterion was that patients have knee articular defects located at the femoral condyles or trochlea, meeting the early OA criteria defined by Luyten et al. [2]: (1) at least two episodes of joint pain for more than 10 days in the last year; (2) radiographic Kellgren–Lawrence classification up to grade 2 (osteophytes only); and (3) arthroscopic findings of cartilage defects (limited to ICRS grade III or IV with softening and swelling of the surrounding cartilage). Exclusion criteria were: (1) primary defects located on the tibial plateau or patella, and (2) patients be affected by osteochondritis dissecans (OCD) or uncorrected lower limb axial defects and knee instability. Conversely, patients undergoing realignment procedures (two high tibial and two distal femoral osteotomies) or ligament reconstruction (four anterior cruciate ligament (ACL) reconstructions and one postero-lateral corner repair) concurrently with scaffold implantation were also included. Patients presenting metabolic, inflammatory, infectious and neoplastic pathologies, including those not able to observe the required post-operative rehabilitation regimen, were excluded from this series.

Twenty-four patients were consecutively enrolled and treated by implanting an osteochondral cell-free scaffold, pursuing a formerly described technique [13]. The short-term results of 23 patients have previously been published [13]. Twenty-two of them (three women and 19 men) were prospectively evaluated before surgery, at 12, 24 and 60 months’ follow-ups, while two patients were lost at the final follow-up and deemed dropouts. Patients were had a mean age of 39.0 ± 8.2 years, and the average body mass index (BMI) was 25.0 ± 2.2. Average lesion size was 3.2 ± 1.9 cm^2^. Five patients had multiple lesions, for a total of 27 defects treated, which were located in 10 cases at the medial femoral condyle (MFC) and in nine cases at the lateral femoral condyle (LFC). Six patients had trochlear lesions, with one tibial plateau and one patella lesion that were included and treated as secondary lesions. Etiology was rated as traumatic (not acute) in five cases and microtraumatic or degenerative in 17 cases. Seven patients were surgically treated for the first time, while 15 patients had undergone previous surgeries: 11 meniscectomy, six anterior cruciate ligament reconstruction, four debridement, four microfracturing and one autologous chondrocyte transplantation, with one treatment of tibial plateau fracture and one loose body removal. Combined surgical procedures were performed in 16 patients: four anterior cruciate ligament reconstruction, two distal femoral osteotomy, two high tibial osteotomy, three meniscal scaffold implantation [two Actifit® scaffold and one collagen meniscus implant (CMI)], two meniscectomy, one loose body removal, one meniscal allograft implantation, one microfracturing and one postero-lateral corner repair.

### 2.1. Surgical Procedure and Patients’ Evaluation

A biomimetic free-cell scaffold with a tri-layered structure that reproduced the osteochondral tissue (MaioRegen, Finceramica SpA, Faenza, Italy) was used for all patients. The superficial layer consisted of type-I collagen and had a smooth surface to mimic the cartilage surface. The intermediate layer (tide mark-like) consisted of a combination of type-I collagen (60%) and hydroxyapatite (40%). Lastly, the lower layer consisted of a mineralized blend of type-I equine collagen (30%) and hydroxyapatite (70%) reproducing the subchondral bone layer. Implantation was executed with a single-step surgical procedure: after the induction of general or spinal anesthesia, the patient put themselves in a supine position with a pneumatic tourniquet around the proximal thigh. The osteochondral procedure was generally the last to be performed in cases of combined procedures. Osteochondral lesions were exhibited through medial or lateral mini-arthrotomic parapatellar approach. The chondral defect was prepared by removing of the injured cartilage and sclerotic subchondral bone using an osteotome, in such a way to create a lodging up to 8-mm deep with perpendicular sides. The scaffold was then shaped and sized according to the prepared lesion site and implanted by press-fit fixation [14]. The stability of the implant was ensured by the deep and rigid shoulders of the prepared osteochondral defect, which is a peculiarity of osteochondral scaffolds compared to other chondral scaffolds. Finally, cyclic flexion-extension of the knee was performed to test the stability of the implant while the graft was visualized, before and after releasing the tourniquet. 

Patients were prospectively evaluated before and after surgery at 12, 24 and 60 months of follow-up. The clinical results were evaluated using the Cartilage Standard Evaluation Form as proposed by the International Cartilage Repair Society (ICRS), which included International Knee Documentation Committee (IKDC) objectives and subjective evaluations. Return to sport was also evaluated with the Tegner score and compared with levels before surgery and before the onset of symptoms. Failures, adverse events and complications were also reported [15,16].

### 2.2. Statistical Methods

The continuous data were reported as the mean and standard deviation of the mean, and the categorical data were expressed in terms of percentages and frequency. Normality of continuous variables was tested with the Kolmogorov–Smirnov test. The differences at different follow-up times were assessed with the repeated measures General Linear Model (GLM), using the Sidak test for multiple comparisons. Differences at the follow-up times of scores that were not normally distributed were evaluated with a Friedman non parametric test, and for multiple comparisons a Wilcoxon post hoc pairwise comparison corrected by Bonferroni method was used. The differences between groups of continuous, normally distributed and homoscedastic data were assessed by an ANOVA test, whereas a Mann–Whitney test was used in the other cases. A Scheffe post hoc pairwise comparison following an ANOVA test was used also to evaluate the differences among groups of normally distributed, continuous and homoscedastic data, a Mann Whitney test with Bonferroni correction for multiple comparisons following a Kruskal–Wallis test was applied in the other cases. Correlations between continuous data were assessed by Spearman’s Rank, and a Kendall’s Tau correlation was used to evaluate the correlation between ordinal data. 

For all tests *p* < 0.05 was considered significant.

All statistical analyses were performed using SPSS v.19.0 (IBM Corp., Armonk, NY, USA).

## 3. Results

During the study follow-ups, no major adverse events were reported. The complication rate of this study was 8.3%, with two cases of joint stiffness treated by knee mobilization under narcosis at two and four months after surgery, respectively, with no further consequences. Four patients failed (16.6%) according to a specific and exhaustive definition including both surgical (two patients, treated with osteochondral autograft transplantation at 24 months’ follow-up in one case and with medial unicompartmental knee arthroplasty at 24 months’ follow-up in the other case) and clinical criteria (two patients) [17]. 

**Figure 1:**
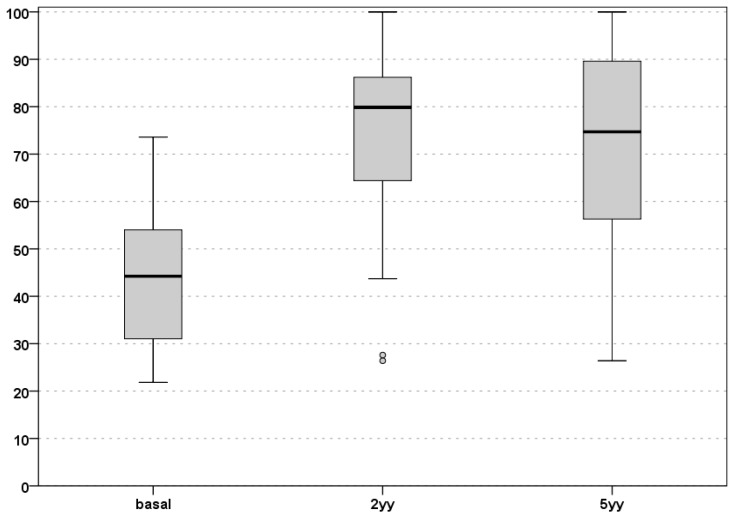
IKDC subjective score evaluation up to 60 months of follow-up. Basal, pre-operative level; 2yy, 2 years’ follow-up; 5yy, 5 years’ follow-up. IKDC: International Knee Documentation Committee.

All scores improved at the two years’ follow-up and remained stable at the final follow-up. In detail, the IKDC subjective score improved from 42.8 ± 13.8 at basal evaluation to 74.9 ± 20.4 at 24 months (*p* < 0.0005), being stable (72.4 ± 22.1, n.s.) up to the final follow-up of 60 months (Figure 1). 

**Figure 2:**
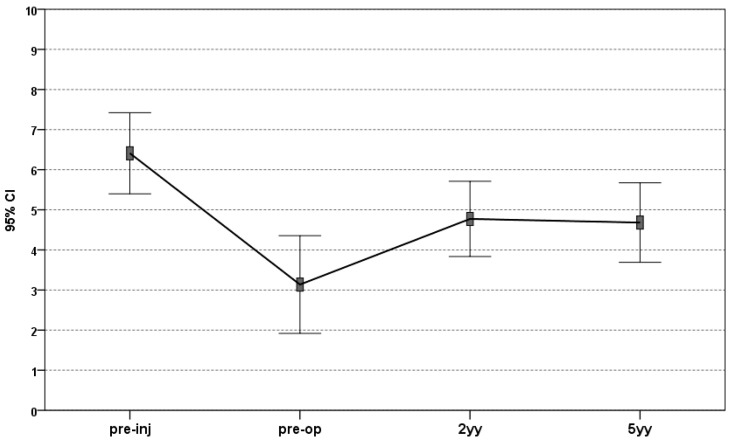
The Tegner score evaluation up to 60 months of follow-up. The score significantly improved from pre-operative level to 2 years’ follow-up, and the score was stable up to 5 years, even if it always remained significantly lower than the pre-injury value. Pre-inj, pre-injury; pre-op, pre-operative; 2yy, 2 years’ follow-up; 5yy, 5 years’ follow-up.

An improvement was also shown via analysis of the IKDC objective score. At basal evaluation, 12 knees (54.5%) were considered “normal” or “nearly normal” (six grade A, six grade B). At the 24 months’ follow-up, a significant increase was documented: 19 knees (86.4%, 14 grade A; 7 grade B) were considered “normal” or “nearly normal”. This evaluation was confirmed at five years’ follow-up (20 “normal” or “nearly normal” knees of which 11 were grade A and 9 grade B). Finally, the Tegner score improved significantly from 3.3 ± 2.7 pre-surgery to 4.7 ± 2.1 at 24 months post-surgery (*p* < 0.005), and remained stable at the final evaluation (4.7 ± 2.2; n.s.). However, the activity level before the onset of symptoms (6.1 ± 2.6; *p* = 0.004) was not reached at any follow-up time (Figure 2).

No significant correlation was found between clinical outcomes and lesions’ or patients’ variables, such as patient’s sex, age, BMI, previous and combined surgery, size of lesion and etiology. Although a higher improvement was observed at two years for patients under 40 years of age, this trend was not confirmed at five years, when no difference could be found.

## 4. Discussion

The main finding of the present paper is that the implantation of cell-free osteochondral scaffold for focal osteochondral defects in early OA knees was safe and offered pain relief and increased function, even in patients with comorbidities addressed concomitantly, with stable results up to five years’ follow-up.

Several reports in the literature had previously shown satisfactory clinical results when this technique was applied for focal chondral and osteochondral defects in non-OA joints [7,8,9,10], and promising results were also observed in more challenging cases, like post-traumatic osteochondral focal lesions of the tibial plateau [11] or as a salvage combined procedure to address unicompartmental OA [18]. Nevertheless, the possibility to successfully treat articular surface lesions in a degenerated environment is still controversial. Regenerative procedures for cartilage repairs, such as autologous chondrocyte implantation (ACI), showed less favorable results when performed in degenerative defects or OA knees, with lower outcomes and a higher failure rate [3,19,20]. A possible explanation relies in the altered joint environment, since inflammatory cytokines present in the joint space may affect the regeneration process by inducing de-differentiation or the apoptosis of the transplanted cells [21]. In fact, synovial inflammation, matrix degradation and changes in the subchondral bone layer can conduct swelling and pain of the joint affected by OA, and can also impair the regenerative potential of intraarticular tissues, as shown by several authors. 

Rodrigo et al. [22] reported the effect of synovial fluid on in vitro chondrocytes cultures, showing that samples from patients with chronically injured knees inhibited chondrogenesis. A preclinical trial published by Saris et al. [23] documented poorer tissue engineered cartilage regeneration in goats when a treatment was performed late after injury. Similarly, Ozsoy et al. [24] confirmed these findings by showing that untreated osteochondral defects led to progressive degenerative alterations in a rabbit model, whereas a normalization occurred after cartilage treatment. Moreover, treatments performed at early stages showed better outcomes compared to delayed ones when chronic degenerative changes were more advanced. However, regenerative procedures for cartilage restoration may still produce positive results in joints affected by degenerative alterations. Hollander et al. [25] reported tissue regeneration after chondral treatment with matrix-associated autologous chondrocyte transplantation (MACT) in OA joints, and laboratory studies have asserted potential regeneration even in this setting [26,27], leaving space for a possible clinical use.

Very few studies have investigated the use of an osteochondral scaffold in OA background. A first study by Marcacci et al. reported satisfactory clinical results when the implantation was performed in unicompartmental OA knees in combination with various procedures aimed at restoring the most appropriate biomechanical environment, such as osteotomies and meniscal substitution [18]. Furthermore, a previous study by Di Martino et al. on the short-term outcome of the current survey showed promising results obtained by this osteochondral approach applied to patients with early knee OA after failed conservative management [13]. Both these studies found better outcomes in patients under 40 years of age. The influence of aging on the body’s regenerative potential is well known, but it should be considered with caution in relation to these treatments. In fact, the present series confirmed a positive effect of younger age at early follow-up, but further analysis found no differences between younger and older patients at mid-term follow-up. This may reflect the fact that older patients require longer times for recovery, or that the influence of age could also be related to a score bias, as shown by a recent study where subgroup differences were not significant after score standardization [28]. In this light, age may represent only a relative limitation for the application of this regenerative approach, and further studies should quantify age influence and its role for treatment indications. Besides a faster recovery in younger patients, the overall group of patients in this study showed an improvement in all scores evaluated two years post-surgery, and were stable up to a mid-term follow-up. 

The results of this study are particularly important due to the mid-term follow-up. In fact, besides a clinical and functional improvement, the ambitious aim of this surgical approach was to delay the need for conventional procedures such as prosthesis implantation, which necessarily require activity restrictions. The mid-term activity level analysis in this series showed significant score improvement compared to pre-surgery levels, even though no complete recovery was observed. The mean final score reflected a limited sport activity, but still an active lifestyle. This is line with other series documenting results of this kind of treatment, and may be partially due to the age of the patient cohort, who were largely too old for most competitive sports. Finally, a complication rate of 8.3% was observed in this study, consistent with those reported in other series [29,30]. Four patients failed: two did not show significant improvement, whereas two others were re-operated on for persistent pain at the treated site. This overall rate of non-responding patients could be considered expected and acceptable for this challenging patient population, especially considering the more burdensome alternative surgical options [3].

This study had some limitations. First of all, the missing of a control group hindered the comparison with untreated patients or patients treated with a concurrent procedure; nevertheless, the promising results of this case series supports the future performance of higher-level comparative studies. The small number of patients partially limited the significance of these results, especially when looking for correlations between patients’ or lesions’ variables and clinical results. However, other series for this peculiar kind of patient have rarely been reported at such follow-up times. Secondarily, several procedures were performed in combination with cartilage reconstruction, making this cohort more heterogenous, but this reflects common clinical practice, since early OA usually presents with a complex onset with different comorbidities that need to be addressed to restore the most favorable conditions for joint regeneration, aiming at an optimal clinical outcome [18,31,32]. This combined biological and biomechanical approach was confirmed to be safe in this study, with no severe adverse events related to the procedure, and comparable outcomes for isolated or combined surgeries. Finally, another significant limitation was the absence of imaging evaluation. The quality of tissue regeneration is one of the most controversial aspects emerging for this osteochondral scaffold, with several studies showing unsatisfactory appearance of the graft [30], despite the slow improvement with time after surgery [7], and a positive overall clinical outcome. Nonetheless, the absence of a correlation between MRI and clinical outcomes is quite common for such cartilaginous procedures [33,34]. Furthermore, there is lack of MRI scoring systems focusing on the peculiarity of osteochondral treatment involving both cartilage and subchondral bone, and future studies are needed to understand the best evaluation method and the possible influence of the imaging findings on clinical outcome. Nevertheless, besides a scientific interest, the imaging appearance of the graft is of secondary interest for such patients, where the clinical improvement and delay of prosthetic procedures remains the main target.

## 5. Conclusions

The implantation of a biomimetic cell-free osteochondral scaffold to treat articular cartilage lesions in early OA knees was shown to be safe with satisfactory outcome, a low complication rate and stable results up five-years’ follow-up. Further studies at longer follow-ups may better define the potential of this approach and its efficacy in delaying more aggressive surgical procedures.
